# Prefronto-Cerebellar Transcranial Direct Current Stimulation Improves Sleep Quality in Euthymic Bipolar Patients: A Brief Report

**DOI:** 10.1155/2014/876521

**Published:** 2014-12-07

**Authors:** Amedeo Minichino, Francesco Saverio Bersani, Francesco Spagnoli, Alessandra Corrado, Francesco De Michele, Wanda Katharina Calò, Martina Primavera, Baoran Yang, Laura Bernabei, Francesco Macrì, Lucilla Vergnani, Massimo Biondi, Roberto Delle Chiaie

**Affiliations:** Department of Neurology and Psychiatry, Sapienza University of Rome, Viale dell'Università 30, 00185 Rome, Italy

## Abstract

*Introduction.* Sleep problems are common in bipolar disorder (BD) and may persist during the euthymic phase of the disease. The aim of the study was to improve sleep quality of euthymic BD patients through the administration of prefronto-cerebellar transcranial direct current stimulation (tDCS). *Methods.* 25 euthymic outpatients with a diagnosis of BD Type I or II have been enrolled in the study. tDCS montage was as follows: cathode on the right cerebellar cortex and anode over the left dorsolateral prefrontal cortex (DLPFC); the intensity of stimulation was set at 2 mA and delivered for 20 min/die for 3 consecutive weeks. The Pittsburgh Sleep Quality Index (PSQI) was used to assess sleep quality at baseline and after the tDCS treatment. *Results.* PSQI total score and all PSQI subdomains, with the exception of “sleep medication,” significantly improved after treatment. *Discussion.* This is the first study where a positive effect of tDCS on the quality of sleep in euthymic BD patients has been reported. As both prefrontal cortex and cerebellum may play a role in regulating sleep processes, concomitant cathodal (inhibitory) stimulation of cerebellum and anodal (excitatory) stimulation of DLPFC may have the potential to modulate prefrontal-thalamic-cerebellar circuits leading to improvements of sleep quality.

## 1. Introduction

Sleep problems are common in bipolar disorder (BD). While it is well established that sleep disturbances are common features during manic and depressive episodes of the disorder, it is now starting to become clear that sleep dysfunctions are also common features in the euthymic phase of the disease [1, 2].

The course of BD has traditionally been viewed as episodic, with symptomatic and functional recovery between mood episodes. This view has recently been challenged by clinical and epidemiological studies that document how, despite symptomatic improvements or recovery following mood episodes, many BD individuals experience cognitive and social impairments even during the euthymic phase [3–5].

Recent studies hypothesized that these impairments could be related to the prefronto-thalamic-cerebellar circuit dysfunctions and, in particular, to the loss of the physiological inverse metabolic activity between dorsolateral prefrontal cortex (DLPFC) (hypoactivity) and subcortical areas such as thalamus and cerebellum (hyperactivity) [6–8]. Therefore, we performed a research study based on the administration of a prefrontal-cerebellar transcranial direct current stimulation (tDCS) protocol to euthymic BD patients in order to improve their neuropsychological functioning.

During the investigation, we noticed an unexpected and remarkable improvement in sleep quality in a large number of patients. We found this to be unexpected and of potential clinical relevance, as both the prefrontal cortex and cerebellum are known to play a role in modulating sleep processes [9, 10] and tDCS has the potential to induce hypomania and insomnia [11]. For these reasons, we performed the present subanalysis specifically centred on assessing the possible effectiveness of prefrontal-cerebellar tDCS on sleep quality.

## 2. Methods

### 2.1. Sample

The sample consisted of 25 outpatients (8 males; 17 females; mean age 41.9 ± 12.62) with a diagnosis of BD Type I (*n* = 15) or II (*n* = 10) in the euthymic phase of the disease (disease duration 17.08 ± 12.05 years). Euthymic phase was defined as having a score of <7 on the Hamilton Depression Rating Scale (HDRS) and a score of <7 on the Young Mania Rating Scale (YMRS). Diagnoses were made through the* Structured Clinical Interview for DSM-IV* Axis I Disorders (*SCID-I*). Patients were consecutively recruited at the Policlinico Umberto I University Hospital of Rome. Patients were screened and excluded for having significant concomitant neurological or organic diseases, comorbid Axis I diagnoses, left handedness, pharmacological treatment with typical antipsychotics, or hospitalization in the last 12 months. All patients were on stable pharmacological treatment with lithium (*n* = 12) and/or anticonvulsants (*n* = 17) and/or atypical antipsychotics (*n* = 17) and/or benzodiazepines (*n* = 10) and/or antidepressants (*n* = 5) for at least two months. tDCS was administered to patients in addition to the standard pharmacological maintenance therapies that they were already receiving; the said maintenance therapies remained unchanged.

### 2.2. tDCS

Brain modulating techniques are nowadays considered safe and effective options for the treatment of several neuropsychiatric conditions, including bipolar disorder; tDCS is a neuromodulating technique that delivers constant low, direct current to the brain area of interest via inhibitory (cathode) and excitatory (anode) electrodes [12–14]. tDCS was applied through two sponge electrodes (surface area = 25 cm^2^) moistened with a saline solution. The electrode montage was as follows: cathodal tDCS on the right cerebellar cortex, 1 cm below and 4 cm lateral to the inion (approximately comparable to the projection of cerebellar lobule VII on the scalp), and anodal tDCS over the left DLPFC; electrode position was determined by the International 10/20 System for EEG Electrodes, such that Fp1 corresponded to the DLPFC. The onset and offset of the intervention involved current being gradually increased and decreased, respectively, over 10 s. The intensity of stimulation was set at 2 mA and delivered for 20 min every working day for 3 consecutive weeks using a Magstim DC Stimulator Plus.

### 2.3. Sleep Assessment

We used the Pittsburgh Sleep Quality Index (PSQI), validated in the Italian language, to assess sleep at baseline and the day after the last tDCS session [15]. The PSQI is a self-administered questionnaire that assesses sleep quality and disturbances over a 30-day period. It consists of 19 self-rated questions grouped into seven clinically derived domains of sleep difficulties (sleep quality, sleep latency, sleep duration, habitual sleep efficiency, sleep disturbances, use of sleep medications, and daytime dysfunction). The seven subcomponents correspond to specific dimensions related to sleep quality that are generally assessed in clinical practice. Each domain is rated equally on a Likert scale that ranges from 0 to 3 and is used to generate a global score of 0–21; global PSQI scores >5 indicate poor overall sleep quality [15].

### 2.4. Statistical Analysis

Data were analyzed trough SPSS software version 20. The normal distribution of data was assessed through the Shapiro-Wilk test. Student's *t*-test was used to compare PSQI scores at baseline and after tDCS treatment. All variables were assessed considering a significance level of 5%.

## 3. Results

PSQI total scores and all PSQI subdomains, with the exception of “sleep medication,” significantly improved after treatment (Table 1).

## 4. Discussion

BD patients (even in euthymic phase) exhibit a significantly worse sleep quality as compared with healthy controls [1, 2]. To the best of our knowledge, this is the first paper in which a positive effect of tDCS on the quality of sleep in euthymic BD patients has been reported.

It has been suggested that sleep, cognitive, and affective dysregulation in BD might be related to overlapping neurobiological systems involving prefrontal cortex and its connections with deeper brain regions [9]. Independent studies, in fact, demonstrated that patients with BD in euthymic states and healthy individuals who underwent sleep deprivation presented similar cognitive and emotional deficits involving the prefrontal cortex [9], supporting the role of prefrontal top-down circuitry in concomitantly modulating cognitive, emotional, and sleep processes. In addition, it is known that the modulation of sleep may represent one of the nonmotor functions of the cerebellum [10, 16]. Knowing this, it is reasonable to hypothesize that concomitant cathodal (inhibitory) stimulation of cerebellum and anodal (excitatory) stimulation of DLPFC may modulate these networks leading to improvements of sleep quality.

Since pharmacological treatment remained unchanged during the stimulation protocol and all patients were on stable treatment for at least two months, this improvement cannot be attributed to the effects of medication. All patients tolerated tDCS without complications, supporting the idea that tDCS may represent an inexpensive, easy to administer, noninvasive, and painless treatment for sleep disturbances in psychiatric patients.

This study has several limitations including the small sample size, the absence of objective biological markers of sleep, and the absence of a placebo control group; in particular, the absence of placebo controls makes it very difficult to assess the impact of the intervention. Despite these limitations, this study represents a starting point in the study of the potential efficacy of prefrontal-cerebellar tDCS to improve sleep quality. Further studies on the topic are warranted.

## Figures and Tables

**Table 1:** PSQI total and subcomponents mean scores before and after treatment.

	Before treatment	After treatment	*P*
Sleep quality	1.52 ± 1.04	0.8 ± 0.76	0.007
Sleep latency	1.28 ± 0.97	0.76 ± 0.59	0.028
Sleep duration	1.36 ± 0.90	0.68 ± 0.55	0.002
Sleep efficiency	1 ± 0.76	0.64 ± 0.48	0.053
Sleep disturbance	1.64 ± 0.95	0.56 ± 0.65	<0.001
Sleep medication	0.84 ± 0.55	0.84 ± 0.55	1
Daytime dysfunction	1.28 ± 0.67	0.56 ± 0.50	<0.001
PSQI total score	8.92 ± 3.30	4.84 ± 2.67	<0.001
